# Improving the quality of patient care in lung cancer: key factors for successful multidisciplinary team working

**DOI:** 10.37349/etat.2024.00217

**Published:** 2024-03-21

**Authors:** Alessandro Morabito, Edoardo Mercadante, Paolo Muto, Anna Manzo, Giuliano Palumbo, Vincenzo Sforza, Agnese Montanino, Claudia Sandomenico, Raffaele Costanzo, Giovanna Esposito, Giuseppe Totaro, Rossella De Cecio, Carmine Picone, Annamaria Porto, Nicola Normanno, Arturo Capasso, Monica Pinto, Maura Tracey, Giuseppe Caropreso, Giacomo Pascarella

**Affiliations:** Pathology Specialist at Hospital Clinic Barcelona, Spain; ^1^Thoracic Medical Oncology, Istituto Nazionale Tumori, IRCCS “Fondazione G. Pascale”, 80131 Naples, Italy; ^2^Thoracic Surgery, Istituto Nazionale Tumori, IRCCS “Fondazione G. Pascale”, 80131 Naples, Italy; ^3^Radiotherapy, Istituto Nazionale Tumori, IRCCS “Fondazione G. Pascale”, 80131 Naples, Italy; ^4^Pathology, Istituto Nazionale Tumori, IRCCS “Fondazione G. Pascale”, 80131 Naples, Italy; ^5^Radiology, Istituto Nazionale Tumori, IRCCS “Fondazione G. Pascale”, 80131 Naples, Italy; ^6^Cellular Biology and Biotherapy, Istituto Nazionale Tumori, IRCCS “Fondazione G. Pascale”, 80131 Naples, Italy; ^7^WSB Merito University in Wroclaw, Fabryczna 29-31, 53-609 Wroclaw, Poland; ^8^Rehabilitative Medicine Unit, Istituto Nazionale Tumori, IRCCS “Fondazione G. Pascale”, 80131 Naples, Italy; ^9^Division of Medical Oncology, Department of Precision Medicine, University of Campania “Luigi Vanvitelli”, 80131 Naples, Italy; ^10^Scientific Directorate, Istituto Nazionale Tumori, IRCCS “Fondazione G. Pascale”, 80131 Naples, Italy

**Keywords:** Multidisciplinary team care, multidisciplinary clinic model, multi-team system, care pathway

## Abstract

International Guidelines as well as Cancer Associations recommend a multidisciplinary approach to lung cancer care. A multidisciplinary team (MDT) can significantly improve treatment decision-making and patient coordination by putting different physicians and other health professionals “in the same room”, who collectively decide upon the best possible treatment. However, this is not a panacea for cancer treatment. The impact of multidisciplinary care (MDC) on patient outcomes is not univocal, while the effective functioning of the MDT depends on many factors. This review presents the available MDT literature with an emphasis on the key factors that characterize high-quality patient care in lung cancer. The study was conducted with a bibliographic search using different electronic databases (PubMed Central, Scopus, Google Scholar, and Google) referring to multidisciplinary cancer care settings. Many key elements appear consolidated, while others emerge as prevalent and actual, especially those related to visible barriers which work across geographic, organizational, and disciplinary boundaries. MDTs must be sustained by strategic management, structured within the entity, and cannot be managed as a separate care process. Furthermore, they need to coordinate with other teams (within and outside the organization) and join with the broad range of services delivered by multiple providers at various points of the cancer journey or within the system, with the vision of integrated care.

## Introduction

Treatment of lung cancer patients is increasingly complex, because of the advanced treatment options and the large number of healthcare providers involved [1]. Additionally, continuous innovation in diagnostic, staging, and treatment options adds complexity to the problem of healthcare delivery [2, 3] and multidisciplinary care (MDC) has been identified as a key factor in the provision of high-quality service for cancer patients [4]. Multidisciplinary team (MDT) meetings have become an indispensable component in oncology care [5] and MDC has been advocated in several international guidelines for lung cancer management in the USA [6, 7], UK [8], Australia [9], and France [10]. Moreover, MDC is one of the objectives outlined by the American Society of Clinical Oncology and the European Society for Medical Oncology [7]. Strategies to encourage collaboration of clinicians and healthcare providers in MDC have also been suggested by the Australian National Service Improvement Framework for Cancer [11] and those issued by the National Comprehensive Cancer Network guidelines for lung cancer [6]. Lastly, MDC in patient management has been recognized as a key performance indicator in lung cancer [12, 13].

Currently, there are two main models of MDC delivery. The most widespread is the multidisciplinary meeting model, in which the patient is evaluated by a team of healthcare experts and the treatment decisions are taken collectively. The second, known as the multidisciplinary clinic model, requires the establishment of a centralized lung cancer clinic, where patients see the appropriate specialists. The multidisciplinary meeting model is frequently planned before or after the multidisciplinary clinic model [14].

This article reviews the existing literature on how to plan an effective MDT by describing its evolution at an organizational level, with a focus on the key determinants that emerge when they work across entity boundaries. The ultimate aim is to provide elements to enhance the treatment path for lung cancer patients by highlighting the barriers and challenges that hinder integrated care services. Promoting a shared vision of roles and responsibilities between MDT groups may be insufficient because interventional support at the multilevel governance could be necessary. The relevant literature search was conducted utilizing different electronic databases (PubMed Central, Scopus, Google Scholar, and Google) by using several terms and free text search, combining words in an appropriate manner (i.e. “MDT barriers”; “MDT communication”; “MDT” + “general practitioner”). The most recent guidelines and recommendations published by regulatory authorities as well as oncology associations were also considered.

## Lung cancer care models

The literature describes three main approaches for lung cancer care: the sequential referral model, the multidisciplinary meeting model, and the multidisciplinary clinic model (Table 1) [2]. The difference depends on if multidisciplinary involvement is continuous throughout the patient pathway or whether it is fragmented or “on demand” [15].

**Table 1:** The main strengths and weaknesses of cancer care models

**Cancer care models**	**Strengths**	**Weaknesses**
Sequential referral model	Standard and well documented model of care Quick decision-making	Reflects only the physician’s point of view Difficulties identifying specialists/providers to refer the patients Often referring physician did not receive the consultation report back from the specialist following the referral The process is not very efficient due to multiple unnecessary referrals which not add value Greater probability of non-adherence to guidelines Waste of time Difficult coordination with other specialists Misdiagnosis or ill-treatment Poor patient satisfaction
Multidisciplinary meeting model	Current model of interdisciplinary care MDT decisions replace the physician’s individual perspective Emphasizes patient-centered care Shorter time-frames from diagnosis to treatment Greater probability of adherence to evidence-based guidelines Careful consideration for inclusion in clinical trials Contributes to the staff’s wellbeing Better communication within the team Improves job satisfaction Helps to identify and manage different MDT risk factors May increase survival rates Improves patient satisfaction and quality of life	Time consuming Possible disagreements and antagonism Difficulty supervising post-discussion care
Multidisciplinary clinics model	Emerging model Promotes coordinated and integrated patient care Rapid access to lung cancer specialists Continuity of care Possibility to evaluate the patient in person Might integrate the services of other professionals (nurses, social workers, pharmacists, etc.) Fewer unnecessary delays from diagnosis to initiation of treatment Better communication among team members Increased diagnostic accuracy Adherence to national/international guidelines Improves clinical and financial outcomes, reducing healthcare costs	Requires a dedicated physical space Some services may be performed by tertiary centres, forcing patients to travel long distances Organizational effort to schedule patient appointments

### Traditional sequential referral model

A referral system describes a dynamic process of communication in which a healthcare professional at one level of the system, having insufficient resources (i.e. equipment, skills, knowledge, drugs) or the relative power to decide their use, requests the assistance of another provider, at the same or higher level, better equipped or specially trained in order to appropriately assist the healthcare needs of a given patient [16–19]. The referral system reflects most healthcare systems worldwide based on the two main types of healthcare structures, namely primary care facilities and hospitals. This organization encourages patients to attempt primary-level care first and then move to a higher level of care as needed [19, 20] while ensuring both the cost-effective uses of hospitals and primary healthcare services [21].

However, referral does not occur in isolation or unilaterally by an individual or an Institution. Rather, it is a formal, intentional, or deliberately planned and organized operationally within the overall healthcare system [18]. For this reason, specific medical guidelines regulate the referral between different healthcare facilities, the roles and responsibilities of various stakeholders, how to choose the best referral location, and where the primary care provider (the original institution or practitioner) can be aided with adequate high-quality patient data and/or information [16]. A successful referral system requires that a patient receives the service in which he was referred, that can only be realized if there is a close relationship among all levels of healthcare professionals [22]. According to the World Health Organization Regional-Office for Europe [16], it requires careful coordination to avoid some potential risks related to (i) system-level and (ii) decision-making problems; (iii) issues with information flow, (iv) process and (v) monitoring; (vi) delays and waiting times. In this model, the patient needs weeks or months to complete the visits ordered by different specialists along the care pathway. However, the chosen treatment plan could lead to inappropriate therapy or result in non-guideline adherent therapies, in addition to low patient satisfaction [23] because patients go to multiple locations at different times during their clinical pathway [2]. Furthermore, clinicians work in isolation rather than in partnership, with patients who perceive a lack of clear communication, wasted time, misdiagnosis, and ill-treatment, which may lead to referrals to other specialists [15, 24].

### Multidisciplinary meeting model

In this model, clinical decisions are defined by a team of medical plus allied healthcare professionals from several disciplines who regularly meet together to evaluate different options, developing an individualized diagnostic or treatment care plan. This is a good model for the delivery of an integrated multispecialty approach in lung cancer, because MDT replaces decisions taken by a single physician, and highlights person-centered care, aiming to ameliorate the patient pathway through communication, collaboration, and more efficient organization of both diagnostics and therapeutic services [25]. It provides the opportunity for healthcare professionals with differing expertise to meet regularly to generate evidence-based decision-making, also considering patient preferences [5]. Many advantages, and some disadvantages, are associated with the multidisciplinary meeting model [26]. MDTs can evaluate diagnostic and treatment options with a decision-making process more adherent to cancer guidelines [27] which could increase patient survival rates [1], although some authors demonstrated that improved care processes due to MDT activity do not necessarily result in better clinical outcomes [3, 27–29]. MDTs also increase patient satisfaction and quality of life. In several studies, patients reported feeling satisfied that a team of experts was caring for them and appreciated better coordination of treatment [4]. Similarly, early integration of palliative care in the team approach appears to contribute significantly to improving quality of life in patients [30]. Furthermore, MDT activity increases consideration for inclusion of patients in clinical trials, promotes access to knowledge about support groups [4, 27], contributes to the well-being of members, and improves job satisfaction [4]. Finally, the team approach helps identify and manage different risk factors that could compromise the achievement of the objectives in the lung cancer meeting [31].

However, several barriers could negatively impact the MDT activities: some are related to the MDT meeting in the strict sense, and others are linked to the implementation of the diagnostic or therapeutic recommendations. The first group of barriers includes staff shortages, non-attendance of one or more MDT members, workload and decision fatigue, lack of sufficient time, because of too many cases scheduled for discussion, as well as case complexity, along with inadequate communication within the team [5, 27, 31–34]. In addition, it has been noted that a lack of technical as well as administrative support could negatively impact the quality of decision-making and may increase negative reactions, such as disagreements as well as antagonism and conflicts [5, 32–34]. The second group of barriers includes inadequate patient data or missing medical records at the meeting, delay in fulfilling the diagnostic test because of a limited number of dedicated slots, postponement of systemic therapy due to an excessive time to final diagnosis, lack of adequate coordination between oncologists and radiation therapists, and insufficient number of chemotherapy sessions [5, 27, 31–35]. Though described as a single cancer team, the different teams and specialists involved in healthcare often do not work, or view themselves, as a single integrated team (as a team of teams) [36]. Ultimately, post-discussion care supervision is required to ensure that any deviation from MDT plans related to patient management does not decrease the overall health benefits [2].

### Multidisciplinary clinic model

A multidisciplinary clinic is a new approach to healthcare delivery. It offers coordinated services for patients aiming to improve early diagnosis and development of appropriate treatment programs, in an attempt to address some of the potential limitations of the MDT meeting [24, 37, 38]. The multidisciplinary clinic model promotes healthcare coordination through the integration of specialists who work in a single clinic space, where team members can communicate and discuss both diagnosis and treatment. This process is important, since it offers easy access to all lung cancer specialists, and ensures continuity of care [15]. However, it requires a physical facility where healthcare consultants deal with patients and their caregivers in real-time [2]. This otherwise known multidisciplinary clinic-based model [15] or group clinic model of MDC [2], involves most activities of an MDT meeting, but the major difference is that the physicians have a chance to evaluate the patient in person, with access to the same laboratory results, images, and data from other specialists. Additional differences are related to the phase of case presentation, the approach to the patient, the participants involved, as well as both the organizational and the healthcare context [37].

Multidisciplinary clinic model uses an integrated approach to plan and deliver cancer services. Typically, clinics incorporate care from multiple specialists and might integrate the relevant activity of other professionals, such as nurses, social workers, and pharmacists. Patients are evaluated by a range of experts, cases are discussed in the MDT meeting and a diagnostic/therapeutic plan is formulated [39]. This multidisciplinary model has been linked to fewer unnecessary delays from diagnosis to treatment initiation, enhanced communication among team members, increased diagnostic accuracy, guided by a review panel of specialists in radiology and pathology, together with adherence to national guidelines. Furthermore, this model improves patient satisfaction [39], as well as clinical and financial outcomes [24] through timely assessment and treatment [40]. Even if organized differently, multidisciplinary clinic models are greatly appreciated by patients, favor doctor-patient communication, and decrease the travel time for clinical appointments and diagnostic tests. Quality of life and patient experience are enhanced with this model while decreasing healthcare expenditures [24]. The multidisciplinary clinic model could be a way to reduce physician burnout because it promotes collegiality and interpersonal relationships, as reported by physicians [40]. The strengths of co-location and co-coordination of care in this model, are contrasted by some generic weaknesses. Patients are sometimes required to go to a tertiary centre for consultations, thus forcing them to travel long distances. If the patient cannot be seen simultaneously by several specialists, referrals and appointments must be organized and planned. This requires a significant amount of organizational, as well as operational, and staffing resources [41, 42].

**Figure 1:**
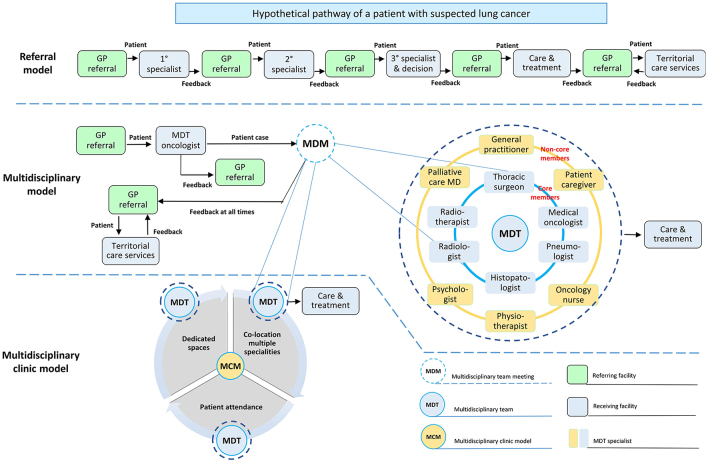
Schematic representation of the main organizational models for lung cancer care. The 1°, 2°, 3° specialist can be an oncologist or a radiotherapist or a thoracic surgeon. GP: General practitioner; MD: medical doctor

The three models described above are summarized in Figure 1. General practitioners are responsible for assessing and managing patient needs and represent the first point of entry for all healthcare models. They play a number of key roles along the cancer care continuum. They initiate the diagnosis and treatment, coordinate patient care as well as referrals to the most appropriate receiving facility [43]. They help manage patient co-morbidities and possible therapeutic complications during treatment. They represent the interface between healthcare professionals of other territorial care services as well as provide necessary information and support to patients and families. Their regular participation in MDC team meetings is encouraged because it favours better coordination of work in regard to therapeutic decisions and ensures a complete care integration: they know their patients, past history, current problems, and desires for the future [44]. However, this approach does not prevent patients from self-referral to specialists when they think necessary, as well as general practitioners who are usually not asked to attend multidisciplinary meetings or have the necessary understanding of this role [43]. In this system, different single referrals to specific care providers (receiving facility) are necessary for the diagnostic phase and the decision about treatment. A single specialist decides what type of intervention is most appropriate for patient needs, including the choice of referring the patient to specialized territorial care services (for example, palliative medical care).

In the multidisciplinary model, patients with suspected lung cancer are seen in the outpatient department by an MDT oncologist who decides if the patient is eligible for discussion during the meeting. In this model, the clinical decisions are made by a team of professionals from several disciplines instead of a single specialist. Since the emphasis on MDC and coordination has increased over time, the multidisciplinary clinic model has been adopted in some centers globally. Three key elements distinguish this model from the multidisciplinary meeting model: (i) the co-location of multiple specialties in the dedicated work-space to facilitate coordination of care and cross-provider communication; (ii) the presence of the patient is encouraged and lastly (iii) availability of dedicated spaces in addition to resources.

## Optimizing pathways for lung cancer patient care

MDT meetings are considered the best practice in management and decision-making for cancer patients worldwide [45], but this does not automatically turn into improved quality of care [46, 47]. Cancer patients utilize a wide range of services from multiple providers at various points during their cancer pathway, requiring integrated care across different organizations and settings over time. However, they frequently experience fragmented healthcare and suffer from a lack of continuity of care [46]. Thus, European, Canadian, and American Cancer Associations are pushing for uniformity in working methods with standardized formats for MDT meetings for optimal coordination and clear communication for patients. Additionally, these meetings are also used for coordinating research, educating, promoting, and diffusing best practices as well as new developments, such as “functional integration” [48]. The implementation of optimal lung cancer care might be a way of improving and reducing variations in patient access to diagnostic and treatment options [46]. Conceptually, optimal lung cancer care must fulfill six factors that ensure high-quality MDT meetings: (i) team approach, leadership, and coordination; (ii) effective and open communication; (iii) full therapeutic range; (iv) standards or guidelines; (v) patient involvement; and (vi) adequate resources. Organizational commitment and infrastructure support are also essential for best-practice approaches to lung cancer. Thus, MDTs require comprehensive management as well as the financial commitment of the institution, in addition to suitable support staff, and an appropriate system for performance monitoring and measurement of these outcomes. The overall process necessitates that lung cancer patients are treated in a comprehensive cancer network made up of different teams along with specialists involved in healthcare, who view themselves, as a single integrated team, to optimally coordinate care across inpatient, outpatient, community, and tertiary settings. The prevalence of multiple chronic conditions may require a multidisciplinary clinical team-of-teams, or a multi-team system, while “transactive memory” could permit the shared division of knowledge held by single team members. Recent innovations in digital health technologies [e.g., electronic health records (EHRs), monitoring, and wearable devices] offer new opportunities in the provision of healthcare services, patient safety, and quality services, described as the transition from Healthcare 1.0 to Healthcare 4.0 [49].

### Team approach and leadership

Optimal patient care cannot be achieved by a single person. Patients need to be managed by multiple healthcare professionals to achieve the best outcome. The composition of the MDT includes “core members” (specialists involved in the key steps of assessment and management of patients with a suspected or confirmed diagnosis of lung cancer) and “non-core or extended members” (a group of professionals from other areas that contribute throughout the process to increase the quality of the service provided) [50]. The team should include healthcare coordinators, medical oncologists, nuclear medicine physicians, nurses (with appropriate expertise), molecular biologists, pathologists, radiation oncologists, radiology imaging specialists, respiratory physicians, and thoracic surgeons. The core team is open to other members or non-core teams, such as clinical psychologists, trial coordinators, dietitians, general practitioners, occupational therapists, palliative care specialists, pharmacists, physiatrists, physiotherapists, psychiatrists, and social workers [26, 47, 50, 51].

Despite various definitions, team leadership can be described as the process of moving or influencing a collection of individuals toward a common objective or vision [52]. The leader of the MDT acts to direct and facilitate discussion, whilst motivating the group to achieve a common goal. Team leadership is critical to facilitate open discussion, including disagreements, allowing the consideration of all opinions across specialties. The role of the chair is to avoid one or more MDT members dominate the meeting by facilitating the participation of all members in the discussion and decision-making phases [53]. However, every meeting has a chair and multiple lead clinicians. Each case will be presented by a physician, usually the one who has seen the patient before the meeting [51].

### Effective and open communication

Cancer care generally requires various interventions provided by multiple healthcare specialists as well as numerous ancillary care professionals, many of them organized territorially in community settings and collaborating across the organizations [54, 55]. They are essential for both diagnosis and treatment, to manage complications, and to ensure continuity of care and adequate patient quality of life throughout the entire oncological experience. Effective and open communication with MDT members, general practitioners, and other secondary care specialists is key for cancer patients [51].

Effective communication requires paying attention to the entire process and not just the content of the message, considering potential barriers at several stages that may prevent the recipient from receiving the message. It is necessary to clarify roles, listen and allow people’s points of view, request the opinions of those who have not spoken, use objective, not subjective language, and be aware of communication barriers (i.e. hierarchy) as well as body language (facial expressions, eye contact, posture), creating an environment of mutual respect avoiding divisive or disparaging language [56, 57]. It improves employee satisfaction and reduces the turnover rate, facilitating the diffusion of a culture based on collaboration, support, and trust because people tend to be more satisfied when they feel they can speak openly [58]. Moreover, open communication facilitates the sharing of best practices, learning from incidents, and the resolution of local issues in the interest of patient care [59]. The positive effects of effective communication within the team naturally promote patient satisfaction in terms of quality care and their family members [53]. Barriers include inter-professional communication, stress, fatigue, team instability, insufficient equipment, as well as inconsistency in leadership. Developing effective communication requires time and continuous commitment [58], inside and outside the whole team. However, the MDT members seldom assess their own communication abilities, and they are usually unaware of what may be considered good communication practices [60] or adequately trained on this topic. Critical issues were also found in communication with patients and other specialists involved in the patient care process. Many patients and family members feel the need for additional information about diagnosis and prognosis, the relevance of routine tests and additional ones that could be requested as well as their usefulness for therapeutic purposes [60]. Communication gaps also characterize MDT relationships with general practitioners and other primary care specialists regarding their ability to provide appropriate advice and coordinate patient care following the multidisciplinary referral, due to inconsistent, delayed, and incomplete information, which includes the lack of a contact person to request relevant or missing data [54].

### Full therapeutic range

The geographical remoteness, small size, and rural or urban healthcare institutions should not impede MDT delivery. All patients, regardless of where they live, should have access to relevant treatments and services. Appropriate systems must be in place to ensure that all patients have access to a wide and comprehensive variety of healthcare options [51, 53]. The availability of a full therapeutic range depends on the relationships with all relevant disciplines for cancer clinical streams [61]. For this reason, the core team could be enlarged (or reduced as needed) to include specialists (i.e. geneticists, psychiatrists, physiotherapists) also from other institutions [32]. Not everyone can be present in the same place and at the same time. So, this requires the identification of local team members and those that need to be connected via the web. Furthermore, the overall objective requires an additional system to enable the flow of knowledge among the MDTs, especially aimed to help small centres that manage a limited number of new cancer patients [53].

### Refer to international standards or guidelines

The diagnosis and treatment of cancer patients should adhere to guideline recommendations [32]. Team meeting decisions need to be evidence-based, patient-centered, and in line with standard treatment protocols [62]. Evidence-based practice guidelines ensure that MDTs deliver high-quality care to patients through the implementation of valid recommendations under specific conditions. Deviation from the prescribed pathway is allowed by guidelines, according to individual circumstances, when the reasons for the deviation can be demonstrated as well as documented. Reasons for not using therapy guidelines include patient circumstances in addition to patient wishes, comorbidities, and preferences, which need to be documented as well as represented by the MDTs with formal mechanisms to put the principles into practice [32].

### Patient involvement

Cancer patients should be encouraged to participate in treatment planning [53], according to a general tendency towards more active patient involvement in healthcare, related to enhanced recognition of their perspective, as well as the right to be involved in decisions concerning their health and healthcare [63]. The team is required to inform the patient about the different treatment options, related risks, and possible complications. Moreover, the patient has to know how to access support services related to the treatment plan and receive all the information regarding ongoing collaboration and communication with team members. Patient involvement improves the quality and centeredness of care and might promote open and participatory communication between both the hospital staff and the patient. This can lead to more trust in services and positive healthcare outcomes, improving therapy adherence in hospice care and the patient’s ability to cope with daily life outside the hospital [53]. It has also been reported that patient involvement does not increase anxiety and it helps to improve both understanding of available treatment choices [26], and patients’ willingness to accept risks for small potential gains [64]. In regards to the healthcare professionals’ perception, patient involvement generates overall satisfaction. On the other hand, studies show that patient attendance during MDT meetings could affect the dynamics of the decision-making process [26, 63], especially in the case of unresolved disagreements about appropriate treatment [32].

### Effective patient follow-up

Lung cancer MDT could improve the management of patients during their treatment, long-term follow-up, and at the end-of-life phase, according to possible transition stages in the cancer care continuum [51]. The rationale for patient follow-up is to evaluate immediate and long-term oncological outcomes, check for recurrences, monitor long-term side effects, and evaluate the opportunity to provide medical support services (physical, mental, and social care needs). At present, the parallel follow-up model (in which cancer-related issues and non-cancer-related care are managed, respectively, by oncology-led and general practitioners) is the most common model and is usually provided in a hospital setting [65]. This model involves different cancer specialists and focuses on surveillance for recurrence but it fails to address many aspects of holistic care [66]. Due to the increase in the number of cancer survivors, the limited workforce, and the inefficient use of the expertise of the oncologist, this model is no longer sustainable [67]. MDT and primary care providers may co-manage many aspects and counsel patients through the whole care process [68]. In particular, the general practitioner has become important for cancer follow-up, but also in the treatment of non-cancer-related health issues, such as comorbidity, toxicity, secondary prevention, health promotion, and coordination of care [67]. This approach to managing the patient after treatment is known as shared or multidisciplinary follow-up. In the broader cancer survivorship care model, shared or multidisciplinary follow-up describes the commitment of both specialists and general practitioners to provide post-treatment care to patients [69, 70] in a formal, explicit manner [71]. This implies an interaction between these specialists in which the key variables are communication through the exchange of information and the definition of mutual responsibilities. Regarding responsibilities, due to medical liability laws, the oncologist maintains the general responsibility and supervises the entire follow-up care process [72].

### Adequate resources

Time dedicated, training, and appropriate resources are required for successful MDT work. Additional time and further resources ensure job satisfaction and reduce staff workloads. Training is important to instruct the team members on the roles and responsibilities of each professional. Training helps to develop all the necessary skills to work as a team, mainly for those who work across organizational as well as professional boundaries [58]. Meetings should preferably be set up in a designated room easily accessible for all participants, with an appropriate layout, in addition to video-conferencing equipped technology for everyone participating in regional as well as supra-regional MDT meetings. Audio-visual equipment should be available to enable tertiary centre participants to review pathology, radiology, or other content adequately and simultaneously [73]. Collaborative MDT working requires an electronic system of medical records to facilitate communication and sharing of patient data, documents, and other pertinent information. With a good HER, providers can ameliorate communication between clinicians, and facilitate access to medical and diagnostic information, in addition to patient history avoiding medical errors [36]. The continuous update of patient data and the possibility of exchanging them with providers, allows the team to make more appropriate diagnostic and treatment decisions improving both patient safety and quality of services. The EHR helps health professionals coordinate patient care in an informed manner at any time and in any place, which allows the expansion and development of the local MDT to a regional or national expert network [74].

## Organizational commitment and infrastructure support

It is widely recognized that developing and sustaining effective MDT working is not a one-off task. The organizations must align mainstream training for MDTs’ needs and support them within the existing infrastructure [75] since MDTs cannot function independently from the established organizational systems [76]. In general, organizational contexts are seen as facilitators for the success of MDTs [77]. This includes several aspects such as technological resources (i.e. electronic communication and EHR), skills and/or capabilities of co-workers, organizational processes (such as care team huddles), quality improvement initiatives, staff training, and supportive and collaborative cultures within their practices [77]. Decision support systems also play an important role (i.e. telemedicine improves meeting attendance and it is cost-effective) [62]. Support at the organizational level is important in the form of protected work time, decreased workload, and adequate data available at the decision-making phase. From the healthcare professionals’ point of view, institutional support for the MDT emerged as a key requirement among doctors and is crucial for the acknowledgment by hospital management about the work-load generated (starting with pre-meeting preparation) and the necessary time for appropriate MDT discussions [78]. As described above, one of the key factors of a successful MDT is resource sharing. To collaborate, each member must be prepared to gather and share their findings with the other team members. It implies that the MDT environment must include an EHR which assists with task switching accordingly, allows good resource communication between members and patients, as well as ensures the availability, confidentiality, and integrity of resources by providing them only to those with proper authorization [79]. Furthermore, the need for a common language of the EHR for MDT meetings has been highlighted, to ensure a centralized database of relevant information such as roles, rational decision-making, and recommended treatment plans [36].

## The MDTs performance measurement system

Similarly like in any line of activity, organizations must recognize and identify their MDTs functioning as well as establish the scope and scale of their practice. Relevant, from this point of view, are the number of functioning MDTs, the time and place in which they meet, the type of training received, how effective MDT support processes have been, as well as the outcomes each MDT achieved [76]. Some of this information and other aspects related to the functioning of these groups (i.e. strengths and weaknesses) can be gathered through a structured survey across operational teams [80], while the measurement of supporting processes and outcomes requires a more comprehensive mechanism to quantify and demonstrate the ongoing results of any MDTs [81]. Key Performance Indicators should be defined in collaboration with all members to ensure consensus, considering that some performance indicators could deteriorate with the introduction of MDTs. An example of this is the case of increased unscheduled hospitalizations linked to more personalized care [58], as well as the increased number of medical tests and workload for diagnostic cost centres. Such circumstances must be considered for performance measurement and evaluation purposes.

## Working in a comprehensive cancer network

MDTs should consider how they could engage with other teams to support individuals with multiple care and social needs. The cancer healthcare system can be envisioned as a multilevel set of factors that affect patients at the different stages of the cancer treatment continuum and various forms of teamwork are executed by different overlapping care providers in this continuum [82]. Furthermore, the prevalence of multiple chronic conditions among lung cancer patients may require a multidisciplinary clinical “team of teams” or a multi-team system [83]. The multi-team system is defined as an “interdependent, highly specialized, and geographically dispersed team of teams involved in the care and health of a particular person” [84] which, ideally, coordinates responsibilities, shares information, and aligns clinical decisions to ensure that comprehensive patient needs are managed [83] across geographic, organizational, and disciplinary boundaries. In cancer care, this “team of teams”, is responsible for coordinating patient healthcare, and often includes physicians, nurses, advanced practice registered nurses, physician assistants, palliative care specialists, clinicians providing psychosocial support, spiritual workers, rehabilitation specialists, pharmacists, hospice clinicians, and others [84]. Boundary spanning is the key between-team processes and can mitigate delays in care. This process encompasses actions of multi-team system members that connect different team components throughout the system to facilitate subsequent processes, such as information sharing across teams to foster a shared understanding among cancer multi-team system components [85]. However, working outside traditional organizational and professional boundaries as part of a multi-team system can sometimes create a sense of loss of professional autonomy [75] and requires a substantial cultural change as well as a system-wide plan that includes recommendations to ensure collaboration at all levels. Joint training helps develop these skills [58], and encourages leadership with a predisposition for collaboration, working with and across boundaries as well as along pathways based on patient needs [86]. Regarding this, overcoming professional cultural barriers represents one of the main challenges of multidisciplinary teamwork. Healthcare professionals must learn to put patients’ interests first to overcome their professional logic and work in a collegial manner [58].

## Transactive memory and the role of information technology

Information Technology is a key element that supports MDT working, particularly when it implements new ways of operating (e.g., multi-team system) because it facilitates information flow among and between teams. This is essential for the decision-making process of members working in different phases of the patient pathway. However, this is an expensive, complex, and time-consuming process to implement [58]. The first strategy to achieve this goal is transactive memory, a process in which “two or more professional teams develop a shared system for encoding, storing, and retrieving information”. Transactive memory can favour the exchange of specialized knowledge held by individual members belonging to different teams and could lead to optimal treatment as well as better coordination of services provided [87]. These results can be further improved by artificial intelligence techniques. Advanced information technologies, new devices, and equipment have the potential to support information sharing and team communication while facilitating data acquisition and patient monitoring [49].

**Figure 2:**
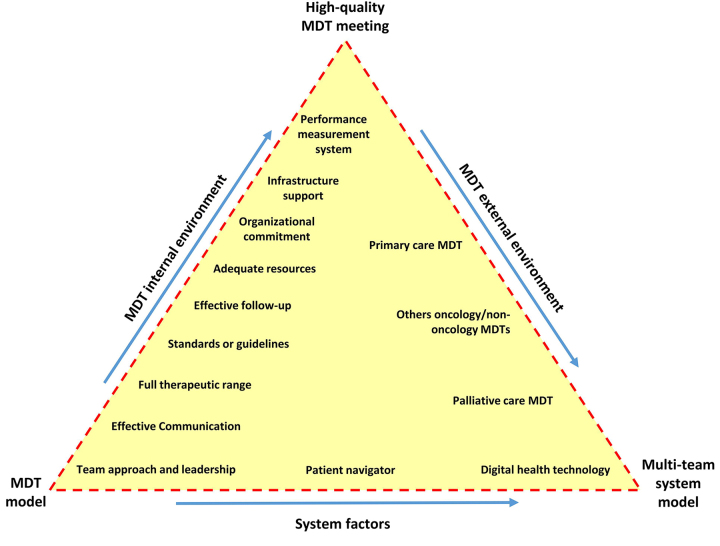
Optimizing pathways for lung cancer patient care

The key factors considered for quality cancer care provision described above are illustrated and summarized in Figure 2. This provides a schematization of the variables that need to be implemented to have a high-quality MDT meeting. These key factors contribute to putting into practice a multi-team system model to work effectively across boundaries. The framework also recognizes the role of the patient navigator [84] alongside digital healthcare technologies. With different weights and relevance, barriers to MDC can hamper the whole system and full implementation of multidisciplinary work at all levels, as also described by Barrios et al. [88]. These authors, discuss the state of the MDC in breast cancer in five Latin American countries, identified a multiplicity of barriers, and proposed possible facilitators for the effective implementation of many key factors as described in Figure 2. They classify barriers into three domains according to their origin: healthcare system, healthcare workers, and patient-related. This confirms the multilevel nature of the barriers.

Key principles that should be implemented to have high-quality multidisciplinary meetings and to ensure the functioning of the multi-team system model are highlighted in the Figure 2. These principles could be grouped in the MDT internal environment (implementation depends on team efforts and/or on the entity they belong to) and external environment (that should be considered when they collaborate within the service territory). The system factors operate at a global level facilitating teamwork beyond organizational boundaries (patient navigator and digital health technology). Each corner of the triangle represents the different stages of the cancer care system. Implementing internal environment factors ensures high-quality MDT meetings, while the multi-team system model requires consideration of external factors. The red dashed line symbolizes, with different weight and relevance, barriers to MDC that may hamper the whole system as well as the development of multidisciplinary work at every level. They must be identified at the organizational and/or national level and adequately managed by different subjects depending on roles and barrier types.

## Conclusions

The MDT is defined as a group of people bound by a common purpose characterized by five elements: (i) shared decision-making, (ii) partnership, (iii) interdependency, (iv) balanced power, and (v) process (development and use of protocols). With the increasing complexity of cancer diagnosis and treatment, MDC and MDT have been identified as cornerstones in lung cancer care. Many key elements of multidisciplinary teamwork appear consolidated, while others emerge as prevalent and actual (i.e. organizational and financial commitment, infrastructure support, performance measurement, and development of a multidisciplinary clinic model), especially those related to the visible barriers when they work across geographic, organizational as well as disciplinary boundaries (for example, the need to have a team of teams, coordination, communication, patient information sharing and multi-team system accountability issues). However, to overcoming barriers to full implementation of MDT care requires a radical cultural change at both the organizational and institutional levels, focused on creating added value to patient care through cross-team collaboration.

## Abbreviations

EHRs: electronic health records
MDC: multidisciplinary care
MDT: multidisciplinary team

## Declarations

### Copyright

© The Author(s) 2024.
